# The Oneiric Activity during and after the COVID-19 Total Lockdown in Italy: A Longitudinal Study

**DOI:** 10.3390/ijerph19073857

**Published:** 2022-03-24

**Authors:** Maurizio Gorgoni, Serena Scarpelli, Valentina Alfonsi, Ludovica Annarumma, Elisa Pellegrini, Elisabetta Fasiello, Susanna Cordone, Aurora D’Atri, Federico Salfi, Giulia Amicucci, Michele Ferrara, Mariella Pazzaglia, Luigi De Gennaro

**Affiliations:** 1 Department of Psychology, Sapienza University of Rome, 00185 Rome, Italy; serena.scarpelli@uniroma1.it (S.S.); valentina.alfonsi@uniroma1.it (V.A.); pellegrinielisa95@gmail.com (E.P.); elisabetta.fasiello@uniroma1.it (E.F.); giulia.amicucci@uniroma1.it (G.A.); mariella.pazzaglia@uniroma1.it (M.P.); luigi.degennaro@uniroma1.it (L.D.G.); 2 Body and Action Lab, IRCSS Fondazione Santa Lucia, 00179 Rome, Italy; ludovica.annarumma@uniroma1.it; 3 Faculty of Medicine, UniCamillus, Saint Camillus International University of Health Sciences, 00131 Rome, Italy; susannacordone@gmail.com; 4 Department of Biotechnological and Applied Clinical Sciences, University of L’Aquila, 67100 L’Aquila, Italy; aurora.datri@univaq.it (A.D.); federico.salfi@graduate.univaq.it (F.S.); michele.ferrara@univaq.it (M.F.)

**Keywords:** dreams, COVID-19, lockdown, pandemic, continuity hypothesis, emotions, sleep, PTSD, disruptive nocturnal behaviors

## Abstract

A growing body of evidence highlights that the COVID-19 pandemic affected oneiric activity. However, only a few studies have assessed the longitudinal changes in dream phenomenology during different phases of the pandemic, often focused on a limited number of dream variables. The aim of the present study was to provide an exhaustive assessment of dream features during total lockdown (TL) and a post-lockdown (PL) period characterized by eased restrictive measures in Italy. We performed a longitudinal study using a web-based survey to collect demographic, COVID-19 related, clinical, sleep, and dream data at TL and PL. Our final sample included 108 participants. The high frequency of poor sleep quality, anxiety, and depressive symptoms observed during TL remained stable at PL, while sleep latency (*t* = −4.09; *p* < 0.001) and PTSD-related disruptive nocturnal behaviors (*t* = −5.68; *p* < 0.001) exhibited a reduction at PL. A PL decrease in time spent with digital media was observed (*t* = −2.77; *p* = 0.007). We found a strong PL reduction in dream frequency (*t* = −5.49; *p* < 0.001), emotional load (*t* = −2.71; *p* = 0.008), vividness (*t* = −4.90; *p* < 0.001), bizarreness (*t* = −4.05; *p* < 0.001), length (*t* = −4.67; *p* < 0.001), and lucid dream frequency (*t* = −2.40; *p* = 0.018). Fear was the most frequently reported emotion in dreams at TL (26.9%) and PL (22.2%). Only the frequency of specific lockdown-related dream contents exhibited a reduction at PL. These findings highlight that the end of the home confinement had a strong impact on the oneiric activity, in the direction of reduced dream frequency, intensity, and lockdown-related contents. The co-occurrence of such changes with a decline in nocturnal PTSD-related symptoms, sleep latency, and time with digital media suggests an influence of post-traumatic stress levels, lifestyle modifications, and sleep pattern on dream changes during different phases of the pandemic. The stable prevalence of fear in dreams and the large frequency of poor sleep quality, depressive symptoms, and anxiety are probably related to the persistence of many negative consequences of the pandemic. Overall, these results are consistent with the continuity hypothesis of dreams.

## 1. Introduction

The COVID-19 pandemic and its consequences dramatically affected psychological well-being [1,2]. Moreover, many studies have documented the strong and complex effects of the pandemic on sleep pattern and their relationship with sociodemographic, environmental, and clinical variables [3,4,5,6,7,8,9,10,11,12,13]. 

Starting from previous observations of dream changes after collective threatening experiences [14,15,16], a growing literature has focused on the phenomenology of the oneiric activity during the pandemic. Several studies conducted in different countries found pandemic-related increases in dream and nightmare recall frequency [17,18,19,20,21,22]. Furthermore, changes in the qualitative features of dreams [19,22,23] and pandemic-related dream contents [24,25,26,27,28,29,30] have been described. Different factors seem to influence specific pandemic-related dream changes [18,19,20,21,22,28,30,31,32], including modifications of the sleep pattern, characteristics of daily experience, emotional status, and sociodemographic variables.

The larger part of the literature on pandemic dreams concerns cross-sectional studies that were performed during the first lockdown and a retrospective evaluation of dream changes compared to the pre-pandemic period. Only a few studies performed a longitudinal assessment of oneiric activity during different phases of the pandemic. Scarpelli and coworkers [33] used sleep and dream diaries to assess the differences between the last week after the first Italian total lockdown (TL) compared to the following week, with the aim of describing the immediate effect of the easing of restrictive measures. The results highlighted a reduction in dream and lucid dream frequency after TL, and increased dream content regarding crowded places and travelling. These changes in oneiric activity co-occurred with greater ease of falling asleep and a reduced number of awakenings after the lockdown. Another Italian study [34] performed a week-by-week evaluation of sleep and dream diaries during the last period of the first TL (29 March 2020–3 May 2020), and a follow-up assessment during a post-lockdown (PL) period, characterized by eased restrictions (October 2020). Beyond reduced sleep latency, greater ease of falling asleep, and higher total bed time at the follow-up examination, an initial increase in dream frequency was observed during the TL, which remained stable until the end of the isolation period, followed by a PL reduction. Conte and coworkers [22] focused on dream modifications during the first and second wave of COVID-19, showing that fewer participants reported qualitative and quantitative dream changes during the second wave. Moreover, dream affect was more negative during both TL (first wave) and partial lockdown (second wave) compared to pre-lockdown periods. In both lockdown periods, greater negative dream emotionality predicted changes in dream quality and frequency and was predicted by poor sleep quality. Finally, Scarpelli and coworkers [32] directly assessed the quantitative and emotional dream features between the first and second pandemic waves in Italy. The results showed that dream, nightmare, and lucid dream frequency were reduced during the second wave compared to the first wave, as well as emotional intensity and nightmare distress. However, dreams had a greater negative emotional tone during the second wave than the first wave. Quantitative and emotional dream features were associated with pandemic-related factors, and the relationship between nightmares and greater risk of post-traumatic stress disorder (PTSD) was highlighted. This association is still stronger in people infected by COVID-19 [35].

Taken together, these results support the notion that the pandemic strongly affected several aspects of the oneiric activity, and that different stages of the pandemic are characterized by changes in dream pattern, according to modifications of the environmental conditions, diurnal emotional status and experiences, and modifications to sleep patterns. In this regard, monitoring dream features during different phases of the pandemic may represent a useful strategy to (a) reach an exhaustive understanding of the impact of the COVID-19 era on dream phenomenology, and (b) provide hints about the main theories about dreaming. Crucially, in accordance with the hypothesis that dreams may represent a reliable index of well-being [32], these findings point to the importance of the assessment of dream features during the evolution of the pandemic. However, among these longitudinal studies, only one [34] focused on dreams during the period of eased restriction between the first and second pandemic waves (represented in Italy by summer and initial part of autumn 2020), which is important to clarify the effect of (total or partial) lockdown periods on dreaming. However, the number of dreams was the only available variable concerning the oneiric activity collected in that study. Moreover, longitudinal data concerning dream contents have been described immediately after the end of the first lockdown [33]. The longitudinal assessment of dreaming activity during lockdown periods and phases characterized by eased restrictive measures would help to distinguish specific lockdown-related changes in oneiric activity during the COVID-19 pandemic.

Starting from these premises, our aim was to provide a thorough assessment of the oneiric pattern during two distinct phases of the pandemic in Italy: the first TL and a following PL period of eased restrictions that preceded the beginning of the partial lockdown that aimed to contain the second pandemic wave. Consistent with the current literature, we expected to find in the PL period, compared to the TL: (a) a reduction in dream frequency and qualitative features, (b) changes in the pattern of dream contents, and (c) the co-occurrence of reduced dream activity with changes in daily habits, better sleep quality and/or improved mental health.

## 2. Materials and Methods

### 2.1. Design

We performed a longitudinal study during the COVID-19 pandemic. The participants’ recruitment was performed via social networks (Facebook, Twitter, Instagram) through a snowball sampling strategy. The study was presented as an investigation on the effect of the forced isolation on dreaming activity and sleep during the COVID-19 pandemic. A total of 1.091 participants completed an online survey during the first COVID-19 Italian TL, from 23 April to 4 May 2020. The results of the cross-sectional study performed on the entire sample during TL have been reported elsewhere [19]. Participants were asked to leave their contact information if they agreed to participate in future phases of the research. Out of the 1.091 participants, 507 left their contact information and were subsequently contacted to participate in a follow-up survey during post-lockdown (PL) Fall (10–28 October 2020), before the beginning of the partial lockdown that aimed to contain the second wave of COVID-19 spread. The survey was shared using Google Forms. Participants filled out the survey after reading the informed consent form, providing their explicit agreement to participate in the research and declaring age ≥18 years. We did not provide monetary compensation to participants. At the beginning of each survey (T1 and T2), participants were asked to provide an alphanumeric code based on specific predetermined criteria that allowed us to match the longitudinal data belonging to each participant. The criteria used to build the alphanumeric code (the first three letters of the parents’ names, day of birth, last three numbers of the cellular phone) were explicitly reported at the beginning of each survey (i.e., T1 and T2) and an example was also reported to help the participant. The inclusion criteria were age of at least 18 years old and having an electronic device available to complete the questionnaire. The exclusion criteria were as follows: participants infected by COVID-19; participants that completed the questionnaire more than one time, as ascertained by the alphanumeric code; report of significant events not related to the pandemic (e.g., pregnancy, abortion, death) during the last month, which were considered potential confounding factors; living outside of Italy. The sample size was estimated using the G*Power 3.1 software [36] for two-tailed *t*-tests (matched pairs) according to data on dream recall frequency from our previous longitudinal study [33], using d = 0.38, α = 0.05, and power = 0.95, which indicated a minimum of 91 participants.

### 2.2. Materials

For each considered timepoint, we collected the following measures:

(a) Demographic and COVID-19 related variables: we administered a questionnaire to collect information about age, gender, education, occupation, Italian area, exposure to COVID-19 at the workplace, cohabitation, having a relative/friend affected by COVID-19, forced quarantine for suspected COVID-19 infection, daily hours spent using digital media.
(b) Anxiety symptoms: we administered the State-Trait Anxiety Inventory (STAI-Y, I-II; [37,38]) to assess anxiety symptoms. This is a self-reported questionnaire, consisting of 40 items: 20 for the evaluation of state-like anxiety (STAI-Y I) and 20 for trait-like anxiety (STAI-Y II). A significant level of anxiety is indicated by scores ≥ 40. In the current sample, the Cronbach’s alpha was 0.95 at TL and 0.96 at PL for STAI-Y I, 0.93 at TL and PL for STAI-Y II.
(c) Depressive symptoms: the Beck Depression Inventory-II (BDI-II, [39,40]), a self-reported questionnaire consisting of 21 items, was used to assess depressive symptoms. A total score > 13 is indicative of depressive disorder. The Cronbach’s alpha was 0.90 at TL and 0.92 at PL in the present study.
(d) Sleep Quality: we administered the Pittsburgh Sleep Quality Index (PSQI; [41]) to assess sleep quality. This is a self-reported questionnaire consisting of 19 items, resulting in 7 subscales (sleep quality, sleep latency, sleep disturbances, use of sleep medications, daytime dysfunction) and a global sleep quality score. A PSQI global score > 5 indicates poor subjective sleep quality. In the present sample, the Cronbach’s alpha was 0.76 at TL and 0.79 at PL. We also used the PSQI-Addendum (PSQI-A; [42]) to evaluate trauma-related subjective sleep disturbances. This is a self-report assessment of seven disruptive nocturnal behaviours commonly observed in individuals with Post-Traumatic Stress Disorder (PTSD): flashes, general nervousness, memories or nightmares of traumatic experience, severe anxiety or panic not related to traumatic memories, bad dreams not related to traumatic memories, episodes of terror or screaming during sleep without full awakening, episodes of acting out dreams (i.e., kicking, punching, running, screaming). A PSQI-A score ≥ 4 is predictive for the discrimination of individuals with and without PTSD [42]. The Cronbach’s alpha was 0.75 at TL and 0.73 at PL in the current study.
(e) Dream features: we collected information on several dream variables referred to the last month. Specifically, we asked the participants to score dream and lucid dream frequency on a 7-point (0–6) Likert scale and different qualitative dream features (i.e., emotional load, vividness, bizarreness, length) on a 6-point (1–6) Likert scale [19,43,44,45,46,47]. Moreover, participants reported the most frequent dream emotion, choosing from happiness, sadness, fear, anger, disgust, pleasure, guilt, shame, surprise. Finally, participants were asked to report the presence of specific dream contents from a list (Table 1) adapted from the Typical Dream Questionnaires (TDS; [48]).

### 2.3. Statistics

The statistical analyses were performed using Statistical Package for Social Sciences (SPSS, version 27, IBM SPSS) and Matlab R2011b. Descriptive analyses were performed to outline sociodemographic, COVID-19-related, sleep, and clinical measures. Variables were expressed as absolute (n) and relative (%) frequency. Mean and standard errors (SE) were reported for continuous variables. 

We performed a McNemar test to assess TL vs. PL differences in the proportion of participants concerning COVID-19 related, sleep, and clinical variables. 

Two-tailed paired *t*-tests were performed to assess changes in dream measures, sleep and clinical variables, and daily time using digital media during TL and PL.

Concerning the emotional tone of the oneiric activity, we calculated the percentage of each emotion reported in dreams during and after the lockdown, providing a descriptive view of the distribution of emotions during each period. Moreover, we divided emotions into positive (Happiness, Pleasure, Surprise) and negative ones (Sadness, Fear, Anger, Disgust, Guilt, Shame). Then, we performed a McNemar test to assess changes in the proportion of positive and negative emotions, and for each considered dream content between the two timepoints.

## 3. Results

### 3.1. Demographic Features

A total of 114 individuals who participated in the web survey conducted during TL completed the follow-up survey. We excluded five participants that completed the questionnaire more than once, and one participant who was reported to be pregnant. The final sample consisted of 108 participants. Table 2 reports the demographic characteristics of the sample.

### 3.2. COVID-19 Related, Clinical, and Sleep Changes

Table 3 reports the proportion of participants, divided according to COVID-19-related, clinical, and sleep variables. About half of the participants referred to themselves as not exposed to COVID-19 due to their job (TL: 51.9%; PL: 47.2%). The larger portion of the sample lived with other people (TL: 86.1%; PL: 87%), had no relative/friend infected by COVID-19 (TL: 85.2%; PL: 80.6%), and was not in forced quarantine for suspected COVID-19 infection (TL: 88.9%; PL: 89.8%). More than 60% of participants reported state and trait anxiety, and 33.3% exhibited depressive symptoms at both timepoints. About half of the sample (TL: 52.3%; PL: 49.1%) exhibited poor sleep quality during and after TL. Disruptive nocturnal behaviors (PSQI-A) were common during the lockdown (TL: 64.5%) but exhibited a decreased frequency at PL (48.1%). The only significant result of the McNemar tests (Table 3) performed on COVID-19-related, clinical, and sleep changes was observed for the PSQI-A: 24 participants that passed from a PSQI-A score ≤ 3 at TL to a score > 3 at PL, while only six participants reported the opposite.

Table 4 reports the results of the direct comparisons (paired *t*-tests) between TL and PL performed on time spent with digital media, sleep, and clinical variables. Time spent with digital media was significantly reduced (*t* = −2.77; *p* = 0.007) at PL. The anxiety and depression scores did not exhibit significant longitudinal changes. Concerning sleep features, we only found significant differences in sleep latency (*t* = −4.09; *p* < 0.001) and disruptive nocturnal behavior (*t* = −5.68; *p* < 0.001), with both showing a reduction at PL. A trend (*t* = −1.76; *p* < 0.08) in the direction of reduced PSQI global score (i.e., better sleep quality) can be observed at PL.

### 3.3. Dream Changes

**Figure 1:**
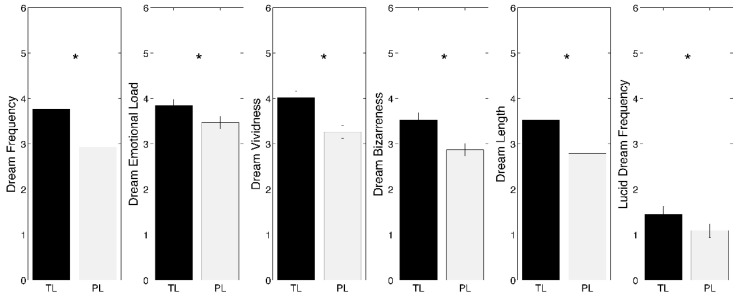
Results of the comparisons (paired *t*-tests) between total lockdown (TL, black bars) and post-lockdown (PL, gray bars), performed on quantitative and qualitative dream variables. Each box represents a dream feature. Error bars represent the standard errors. Asterisks index significant differences (*p* < 0.05).

Figure 1 depicts the comparisons (paired *t*-tests) between TL and PL performed on quantitative and qualitative dream features, showing a drastic reduction at PL in dream frequency (*t* = −5.49; *p* < 0.001), emotional load (*t* = −2.71; *p* = 0.008), vividness (*t* = −4.90; *p* < 0.001), bizarreness (*t* = −4.05; *p* < 0.001), length (*t* = −4.67; *p* < 0.001), and lucid dream frequency (*t* = −2.40; *p* = 0.018).

**Figure 2:**
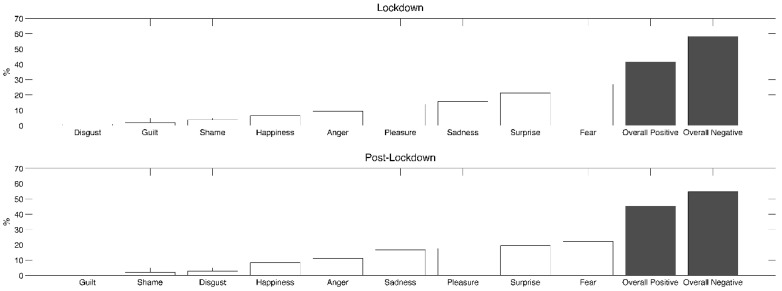
Percentage of each assessed emotion (white bars) reported in dreams during total lockdown (upper box) and post-lockdown (lower box). Black bars represent the overall percentage of positive and negative emotions in each time period.

Figure 2 shows the distribution of dreams’ emotional tone at TL and PL. At both timepoints, fear (TL: 26.9%; PL: 22.2%) and surprise (TL: 21.3%; PL: 19.4%) were the most frequent emotions reported in dreams. Negative emotions were more frequent than positive emotions at both timepoints (TL: 58.3%; PL: 54.6%), without observable significant changes using the McNemar’s test (*p* = 0.62).

**Figure 3:**
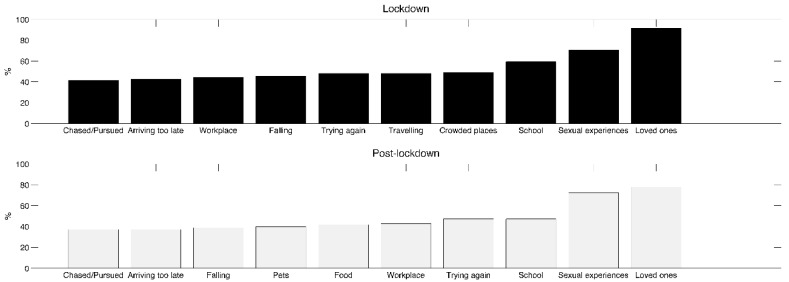
Frequency of dream contents. The bars represent the 10 most frequently reported dream contents during total lockdown (upper box) and post-lockdown (lower box).

The 10 most frequent dream contents are reported in Figure 3. At both TL and PL, the most frequently reported dream contents were loved ones (TL: 91.7%; PL: 77.8%), sexual experiences (TL: 70.4%; PL: 72.2%), and school/teachers/studying (TL: 59.3%; PL: 47.2%). Results of the McNemar’s tests performed on dream contents (Table 5) point to the PL period showing a decrease in the proportion of the following themes: being frozen with fright (*p* = 0.04), being isolated/locked up/shut down (*p* = 0.03), being half awake and paralyzed in bed (*p* = 0.004), loved ones (*p* = 0.001), being in crowded places (*p* = 0.03), war (*p* = 0.007), and traveling (*p* = 0.01).

## 4. Discussion

In the present study, we provide an exhaustive assessment of dream features during TL due to the COVID-19 pandemic compared to the PL period without home confinement using a web-based survey in an Italian sample. Our main findings show that: (a) dream frequency, emotional load, vividness, bizarreness, length, and lucid dream frequency exhibited a drastic reduction in PL; (b) the emotional tone of the oneiric activity remained substantially stable between TL and PL, with a greater percentage of negative affect and fear as the most frequently reported emotion in dreams; (c) only specific dream contents exhibited longitudinal changes in their frequency after TL, all in the direction of a reduction at PL; (d) the large frequency of self-reported poor sleep quality, anxiety, and depressive symptoms remained stable in the considered timepoints; (e) sleep latency, disruptive nocturnal behaviors, and daily time spent using digital media exhibited a reduction at PL compared to TL. 

### 4.1. Quantitative and Qualitative Aspects of Dreams

The observation of a drastic reduction in quantitative (i.e., frequency) and qualitative (i.e., emotional load, vividness, bizarreness, length) dream features in PL is consistent with previous findings, pointing to an intensification of dream frequency and intensity in the TL period during the first pandemic wave [17,18,19,20,21,22,32,33]. In particular, this finding is consistent with our previous retrospective result of increased dream frequency, emotional load, vividness, bizarreness, and length during TL compared to the pre-pandemic condition [19], suggesting that the large changes in dream frequency and intensity observed during the first COVID-19 wave were mainly related to the lockdown condition, and the end of the home confinement seems to have “renormalized” the quantitative and qualitative aspects of dreaming activity. As previously observed [19], the simultaneous and consistent modifications to the frequency and intensity of the oneiric activity may be due to the strong association between the quantitative and qualitative facets of dreams. On one hand, the salience of dreams is considered an important determinant of dream recall frequency [49]. Therefore, a reduction in the subjective impact of dreams may result in decreased dream recall. On the other hand, a decreased number of recalled dreams may lead to a subjective perception of reduced dream salience.

Concerning the decreased lucid dream frequency at PL, our finding is consistent with the observation of reduced lucid dreams during the first week after TL (assessed with dream diaries) [33] and the second pandemic wave (assessed with the Mannheim Dream Questionnaire) [32] compared to TL. Taken together, the results from these studies point to a stable reduction in lucid dream production at different timepoints (with different levels of restrictive measures) after TL. The lucid dream represents a condition in which an individual is aware of his dream while remaining asleep [50]. It has been proposed that such a phenomenon may play a role in the emotional regulation of people experiencing adverse events [51]. In this regard, it could be speculated that greater lucid dream frequency during TL may represent an attempt to enhance coping abilities during the first pandemic wave, a process that may be less involved in the following phases of the pandemic. An alternative explanation (not mutually exclusive) is that the reduction in lucid dreams represents a byproduct of the generalized reduction in dreaming activity.

### 4.2. Dream Contents

Previous studies have highlighted that the pandemic had an influence on dream and nightmare contents [18,25,26,27,28,29,30]. Here, we found that, despite a substantial stability in the most frequently reported dream themes, only the frequency of specific dream contents was reduced in PL. In particular, a decrease proportion was observed in the following contents: being frozen with fright, being isolated/locked up/shut down, being half awake and paralyzed in bed, loved ones, being in crowded places, war, travelling. Our findings are consistent with the “continuity hypothesis” of dreaming activity, which assumes that dreams reflect waking experiences, emotions and mental activity [52,53], suggesting a continuum between waking and sleeping neurobiological functioning and mental activity [51,54]. It has been proposed that dream content mirrors components of daily experience [55,56] and exhibits a preferential response to present worries and events with high emotional charge and personal meaning [57,58]. Indeed, increased bad dreams and nightmares have been observed during stressful periods [59,60]. Therefore, it is plausible that the selective decreased frequency of specific dream contents at PL observed in the present study reflects a reduced emotional charge of specific concerns and personal meanings associated with the experience of home confinement during TL. Indeed, two of these themes (being frozen with fright, being half awake and paralyzed in bed) can be considered the expression of a general topic of paralysis [30] associated with a fearful condition: one (being isolated/locked up/shut down) seems strictly coherent with the TL experience, one (war) appears evocative of an exceptional and dangerous context, and three (being in crowded places, travelling, loved ones) concern objects and experiences that were strongly limited during TL. Overall, such themes may represent specific lockdown-related topics. Of note, themes more explicitly associated with COVID-19 (e.g., being infected by a virus; pandemic/epidemic) did not exhibit changes in their frequencies, consistently with the continuity hypothesis (i.e., during the PL period, the pandemic was still ongoing). However, it should be noted that the mechanisms and timing of incorporation of specific contents in dreams is still poorly understood, and dream imagery seems to incorporate the personal meaning of the experience instead of explicit episodic memories.

Our finding of a reduction in dreams about crowded places and travelling at PL appears to be at odds with our previous result of an increase in the frequency of these dream themes at the end of home confinement [33]. Beyond the methodological differences between these studies (web-based survey in the present study vs. dream diaries in the previous study), it should be considered that this result was obtained by comparing very brief and close periods (i.e., the last week before and the first week after the end of TL). It could be speculated that the time spent in a specific condition influences the emotional relevance of particular experiences and, in turn, the frequency of dream themes associated with such experiences. The restrictions imposed on travelling and being in crowded places during the TL would have represented a very stressful condition, which may have frequently been incorporated in dreams. Similarly, the possibility of going out, meeting other people and restarting a partially ordinary routine immediately after the long period of isolation may have represented an experience with a strong emotional valence for many individuals, triggering the greater frequency of dreams concerning crowded places and travels that were observed in our previous study [33]. On the other hand, after about five months of eased restrictions compared to the period of home confinement (i.e., the PL period considered in the present study) the emotional relevance of these topics may have declined, resulting in a reduced incorporation in dreams compared to TL. 

### 4.3. Dream Emotional Tone

Despite the observed changes in dream quantity, quality, and content, we did not find significant modifications in the emotional tone of dreams, with a greater percentage of negative emotions and fear as the most frequently reported emotion at both timepoints. During TL, compared to the pre-TL period, we previously found an increase in negative emotions in dreams [19]. Moreover, fear was the most frequently reported emotion in dreams, while, in the pre-TL period, surprise was the most frequent emotion, followed by pleasure [19]. Conte and coworkers [22] found an increase in dreams’ negative emotionality in both total and partial lockdown periods (respectively, first and second COVID-19 waves) compared to the periods before them, suggesting a close relationship between dream emotionality and lockdown-related changes in lifestyle and emotional experience. Another Italian study reported more negative emotional valence of dream experience during the second wave compared to the first TL [32]. A reduced adaptive capacity was observed in the face of the second pandemic wave [61] and it has been proposed that greater negative emotional tone may represent an indicator of poorer resilience [32,62]. Differing from these previous studies [22,32], we did not assess the level of negativity/positivity of dream emotional tone but the frequency of specific negative/positive emotions. In this regard, considering the available findings and the results of the present study, it is reasonable to hypothesize that, while the negative emotional valence oscillates during different phases of the pandemic (as highlighted by previous studies), the most frequently reported emotions remain relatively stable during the pandemic, with a greater proportion of fear. It should be considered that, beyond changes in the restrictive measures, during different phases of the pandemic, people remained exposed to several threats and stressful conditions (e.g., risk of contagion, partial limitations of social bonds, economical and working issues) that may affect waking and dreaming emotionality. 

### 4.4. Sleep Pattern, Clinical Measures and COVID-19 Related Variables

Beyond changes in dreaming activity, in the present study, we assessed longitudinal modifications in sleep, clinical measures, and diurnal habits. This choice was made with the aim of controlling for possible changes in daily experience and sleep pattern that may affect dreaming according to previous findings [18,19,20,54]. Interestingly, the observed longitudinal modification in the phenomenology of the oneiric activity occurs in the absence of substantial changes in anxiety and depressive measures, and the frequency of anxiety and depressive symptoms remains high at PL. Concerning sleep, the frequency of poor sleep quality was high at both timepoints, and the global sleep quality score does not exhibit significant longitudinal changes, although there is a trend in the direction of better sleep quality at PL. Considering specific sleep variables, only the reduction in PTSD-related disruptive nocturnal behaviors and sleep onset latency at PL was observed. Taken together, these results are consistent with previous findings showing that, beyond a decline in stress measures at PL, several negative consequences of the pandemic on mental health and sleep persisted after the end of the TL [12,63,64,65]. Although we did not directly assess diurnal stress variables, the disruptive nocturnal behaviors measured by the PSQI-A are commonly observed in individuals with PTSD. Indeed, the PSQI-A score can discriminate individuals with and without PTSD [42]. Therefore, the PSQI-A score represents a reliable measure of post-traumatic stress level expressed during sleep. In this view, our findings on clinical and sleep measures can provide a possible speculative interpretation to the present results on oneiric activity. Indeed, the reduction in stress levels at PL, expressed during sleep by disruptive nocturnal behaviors, may drive the weakening of dream frequency, intensity and specific negative contents. In other words, considering that bad dreams and nightmares are part of the disruptive nocturnal behaviors measured by PSQI-A, the observed PL changes in dreams phenomenology should mainly represent a reduction in the stress level expressed during sleep, most likely associated with the previously documented drop in diurnal stress [12,64,65]. On the other hand, the persistence of a greater proportion of fear in dreams during the PL period may mirror the persistence of the high frequency of depression and anxiety. Such an interpretation would be consistent with the “continuity hypothesis”. Moreover, it could be suggested that the reduced sleep latency and disruptive nocturnal sleep behaviors at PL, together with the observed trend of better sleep quality, may represent indirect indices of a lower level of physiological arousal before and during sleep. In this view, the co-occurrence of these changes in the sleep pattern with the reduction in qualitative and quantitative aspects of dreaming activity would be consistent with the hypothesis that a greater level of arousal promotes dream recall [54]. However, in the absence of objective sleep measures, this remains a speculation.

According with the “continuity hypothesis”, another point that should be considered is represented by the environmental and lifestyle changes at PL. In the present study, no significant longitudinal modifications were observed in the frequency of specific COVID-19-related environmental variables, but we found a PL decrease in the daily time spent using digital media. Interestingly, a recent study found a relation between dream contents and time spent using social media [66]. In this view, and considering the crucial role that digital media has had during the lockdown in our society, it is possible that the strong reduction in the time spent using digital media may have had an influence on dreaming activity. This hypothesis should be directly investigated in future research. Unfortunately, we did not collect much environmental and lifestyle information that could have had a potential influence on dreams. As an example, a reduction in time spent in home was recently found during the first month after lockdown compared to the TL in Italy, but both timepoints showed a higher number of hours-per-day spent in home than in the pre-TL period [12]. These kind of changes in daily routine should be considered in future studies on dreams during the COVID-19 era.

From a broader perspective, together with the growing number of studies conducted during the pandemic, the present results provide further evidence about the strong impact of threatening collective experiences on dreams and sleep. Sleep disturbances are common after potentially traumatic events, such as natural disasters [67,68,69,70,71], marine explosion [72], and combat [73,74,75], and can be long-lasting [68,69,71,72,73]. Moreover, changes in dreaming activity have been reported after experiences such as earthquakes [14] and terroristic attacks [16]. The intrinsic characteristics of the COVID-19 pandemic (i.e., long duration, global nature, periodic changes in countermeasures to prevent the virus) offer a unique opportunity to increase our understanding of sleep and dream modifications in response to collective trauma.

### 4.5. Limitations

Several limitations of the present study should be considered in relation to the generalizability of the results. As previously observed (e.g., [11,12,19], the online recruitment strategy may introduce an issue of partial self-selection, attracting individuals with sleep or mental health problems, or people with greater interest in dreams. Moreover, our sample was unbalanced for several variables and cannot be considered representative of the Italian population. Indeed, our sample was characterized by a greater prevalence of females (76.9%), which represents a common issue in many studies conducted during the pandemic (e.g., [5,11,12,19,20,76]), and almost the entire sample (99.07%) had at least a high school degree. The imbalance concerning these variables may have had an impact on the present findings. In particular the existence of sex differences in dream features [77,78], insomnia [79], and emotional reaction to negative stimuli [80,81,82] should be considered. Furthermore, concerning education, Schredl and Bulkeley [18] found pronounced negative effects of the pandemic on dreaming in individuals with higher education levels. Therefore, great caution should be taken when interpreting these results. 

Compared with other cross-sectional surveys during the pandemic, the present longitudinal study was characterized by a smaller sample size, limiting the generalizability of our findings to the population at large. It should be noted that we did not send reminders to the individuals after inviting them to participate in the second survey, likely limiting the number of participants. Our final percentage of respondents at the follow-up (i.e., 21.3%) is similar to other longitudinal sleep studies during the pandemic [9,34,83].

Another limitation of the present study is represented by the absence of information regarding pre-pandemic sleep and mental health, as well as objective sleep measures. Moreover, we highlighted the absence of measures regarding diurnal stress and PTSD symptoms, which are only assumed by PTSD-related disruptive nocturnal behaviors. The absence of a comprehensive psychopathological and sleep assessment limits the possibility of controlling for the influence of pre-existing mental illness (particularly pre-pandemic traumatic symptomatology) and sleep problems on dream features. Finally, it is worth noting that the PL period was characterized by an increase in the COVID-19 contagions, and we cannot account for the possible effect of the increased pressure of the infections on dreaming. 

## 5. Conclusions

The present study adds a novel piece of knowledge about the characterization of dreaming activity during the COVID-19 pandemic. In particular, we highlighted that several peculiarities of the oneiric activity observed during the COVID-19 pandemic are directly related to the lockdown condition. Indeed, consistently with the continuity hypothesis, we found that a period without home confinement, compared to the TL, was characterized by reduced dream frequency and intensity, lucid dream frequency, and specific dream contents that were likely associated with the condition of isolation. Such changes in dreaming activity co-occurred with a reduction in PTSD-related nocturnal disruptive behavior, sleep latency, and diurnal digital media usage. On the other hand, the predominant emotional tone of dreams remained mainly characterized by fear. Moreover, depression, anxiety symptoms and poor sleep quality were stably high at both timepoints.

Further investigations are needed to clarify the relation between changes in diurnal experiences and dream phenomenology during different phases of the pandemic, with the aim of identifying reliable emotional, environmental, and sleep-related mediators.

In the general framework of the continuity hypothesis, the observation of a strong influence of the lockdown on different facets of oneiric activity suggests that dreams may represent a viable means of access to the individual emotional experience and the process of meanings’ construction during the pandemic, emphasizing the possible role of dreams in a clinical context for the promotion of mental health during the COVID-19 era.

## Figures and Tables

**Table 1:** Categorization of dream contents.

Dream Contents	
1. Being chased or pursued, but not physically injured	32. Fire
2. Being injured	33. A person now dead as alive
3. Being physically attacked (beaten, stabbed, raped, etc.)	34. A person now alive as dead
4. Trying again and again to do something	35. Failing an examination
5. Being frozen with fright	36. Suffocation, breathing problems
6. Food, eating	37. Feral and violent animal
7. Arriving too late, e.g., missing a train	38. Pandemic/epidemic
8. Swimming	39. Being at a movie/cartoon/videogame/comic book
9. Being isolated/locked up/shut down	40. Killing someone
10. Pets	41. Lunatics or insane
11. Money	42. Being half awake and paralyzed in bed
12. Flying or soaring through the air	43. Seeing a face very close to you
13. Falling or being on the verge of falling	44. Seeing and UFO or an extra-terrestrial
14. Being inappropriately dressed	45. Being an animal
15. Being nude	46. Being a child again
16. Being tired, unable to move	47. Seeing an angel or encountering God in some form
17. Being infected by a virus	48. Discovering a new room at home
18. Having superior knowledge, superpowers or magic abilities	49. Airplane crash
19. Seeing him/herself in the mirror	50. Someone having an abortion
20. Natural disasters (earthquakes, floods, tornados…)	51. Being sick
21. Insects, spiders or snakes	52. Being close to some sick
22. Being a member of the opposite sex	53. Zombies
23. Being an object (e.g., tree or rock)	54. Dictatorship
24. Encountering a kind of evil force, monsters or demon	55. Being betrayed
25. Your teeth falling out/losing your teeth	56. Being at the workplace
26. Being killed or seeing yourself as dead	57. Loved ones (family, friends)
27. Vividly sensing, but not necessarily seeing or hearing, a presence in the room	58. Being in crowded places (restaurants, clubs, concerts…)
28. Being unable to find, or embarrassed about using a toilet	59. War
29. School, teachers, studying	60. Travelling
30. Sexual experiences	61. Social media interactions (video calls, chats, …)
31. Losing control of a vehicle	62. Be possessed

**Table 2:** Demographic characteristics of the sample.

	Overall Sample (*n* = 108)	
	N	%
*Gender*		
Male	25	23.15
Female	83	76.85
*Age (Mean ± SE: 32.12 ± 1.29 years; range: 18–88)*		
18–24	42	38.89
25–29	22	20.37
30–39	22	20.37
>40	22	20.37
*Education*		
Middle school	1	0.93
High school	33	30.55
Undergraduate/Graduate	56	51.85
Post-graduate	18	16.67
*Occupation*		
Student	47	43.52
Employed/Self-employed	47	43.52
Unemployed	9	8.33
House husband/wife	3	2.78
Retired	2	1.85
*Italian area*		
North	14	12.96
Center	67	62.04
South	27	25.00

**Table 3:** Proportion of participants concerning COVID-19-related, sleep, and clinical variables during (TL) and after (PL) the lockdown. Results (*p*-values) of the McNemar tests were also reported. The asterisk indexes a significant difference (*p* < 0.05).

	Total Lockdown (TL)		Post-Lockdown (PL)		*p*
	N	%	N	%	
**COVID-19 related features**					
*COVID-19 exposure at the workplace*					0.19
Not employed	31	28.71	29	26.85	
Not Exposed	56	51.85	51	47.22	
Exposed	21	19.44	28	25.93	
*Cohabitation*					1.00
Alone	15	13.89	14	12.96	
With others	93	86.11	94	87.04	
*Knowing a relative/friend infected by COVID-19*					0.33
Yes	16	14.81	21	19.44	
No	92	85.19	87	80.56	
*Forced quarantine for suspected COVID-19 infection*					1.00
Yes	12	11.11	11	10.18	
No	96	88.89	97	89.81	
**Sleep and clinical features**					
*PSQI Global ^a^*					0.60
PSQI ≤ 5	51	47.66	55	50.93	
PSQI > 5	56	52.34	53	49.07	
*PSQI-A ^a^*					* 0.001
PSQI-A ≤ 3	38	35.51	56	51.85	
PSQI-A > 3	69	64.48	52	48.16	
*STAI-I*					0.54
STAI-I ≤ 39	35	32.41	39	36.11	
STAI-I > 39	73	67.59	69	63.89	
*STAI-II*					0.38
STAI-II ≤ 39	33	30.56	38	35.18	
STAI-II > 39	75	69.44	70	64.82	
*BDI*					1.00
BDI ≤ 13	72	66.67	72	66.67	
BDI > 13	36	33.33	36	33.33	

^a^ Calculated on 107 and 108 participants during and after the lockdown, respectively.

**Table 4:** Results of the comparisons (paired *t*-tests) between total lockdown (TL) and post-lockdown (PL) clinical, sleep, and digital media usage measures. Mean and standard errors (SE) are reported. Asterisks index significant differences (*p* < 0.05).

	Total Lockdown (TL) Mean ± SE	Post-Lockdown (PL) Mean ± SE	t_107_	*p*
Time with digital media (h/d)	7.81 ± 0.29	7.03 ± 0.29	−2.77	0.007 *
STAI-I	46.45 ± 1.15	46.19 ± 1.30	−0.24	0.81
STAI-II	44.86 ± 1.11	44.81 ± 1.11	−0.06	0.95
BDI	11.94 ± 0.85	11.33 ± 0.92	−0.72	0.47
PSQI Global score	6.58 ± 0.33	6.04 ± 0.33	−1.76	0.08
PSQI C1 Sleep quality	1.25 ± 0.07	1.17 ± 0.06	−1.13	0.26
PSQI C2 Sleep latency	1.34 ± 0.10	0.95 ± 0.08	−4.09	<0.001 *
PSQI C3 Sleep duration	0.79 ± 0.08	0.91 ± 0.09	1.40	0.16
PSQI C4 Habitual Sleep Efficiency	0.76 ± 0.09	0.61 ± 0.09	−1.66	0.10
PSQI C5 Sleep disturbance	1.27 ± 0.06	1.21 ± 0.05	−1.35	0.18
PSQI C6 Sleeping Medication	0.22 ± 0.07	0.22 ± 0.07	0.00	1.00
PSQI C7 Daytime dysfunctions	0.94 ± 0.07	0.97 ± 0.07	0.38	0.71
PSQI-A	5.63 ± 0.37	3.96 ± 0.31	−5.68	<0.001 *

**Table 5:** Dream contents exhibiting a significant difference (McNemar test) between total lockdown (TL) and post-lockdown (PL). For each dream content, we provide the following information: percentage of individuals reporting the specific dream content at TL and PL; TL vs. PL difference (TL–PL); number of participants exhibiting appearance, disappearance or unchanged presence of the specific content at PL; *p*-value at the McNemar test.

	Being Frozen with Fright	Being Isolated/ Locked Up/ Shut Down	Being Half Awake and Paralyzed in Bed	Loved Ones	Being in Crowded Places	War	Traveling
TL (%)	32.41	28.70	25.00	91.67	49.07	17.59	48.15
PL (%)	22.22	17.59	12.04	77.78	35.19	7.41	32.41
Change (%)	−10.19	−11.11	−12.96	−13.89	−13.88	−10.18	−15.74
Appearance at PL (*n*)	7	7	4	3	14	2	11
Unchanged (*n*)	83	82	86	87	65	93	69
Disappearance at PL (*n*)	18	19	18	18	29	13	28
McNemar (*p*)	0.04	0.03	0.004	0.001	0.03	0.007	0.01

## Data Availability

The data presented in this study are available on request to the corresponding author.
